# Non-Destructive Volume Estimation of Oranges for Factory Quality Control Using Computer Vision and Ensemble Machine Learning

**DOI:** 10.3390/jimaging11100352

**Published:** 2025-10-09

**Authors:** Wattanapong Kurdthongmee, Arsanchai Sukkuea

**Affiliations:** School of Engineering and Technology, Walailak University, 222 Thaibury, Thasala, Nakorn Si Thammarat 80160, Thailand; arsanchai.su@wu.ac.th

**Keywords:** machine learning, computer vision, non-destructive testing, stacking, ensemble learning, quality control

## Abstract

A crucial task in industrial quality control, especially in the food and agriculture sectors, is the quick and precise estimation of an object’s volume. This study combines cutting-edge machine learning and computer vision techniques to provide a comprehensive, non-destructive method for predicting orange volume. We created a reliable pipeline that employs top and side views of every orange to estimate four important dimensions using a calibrated marker. These dimensions are then fed into a machine learning model that has been fine-tuned. Our method uses a range of engineered features, such as complex surface-area-to-volume ratios and new shape-based descriptors, to go beyond basic geometric formulas. Based on a dataset of 150 unique oranges, we show that the Stacking Regressor performs significantly better than other single-model benchmarks, including the highly tuned LightGBM model, achieving an $R^{2}$ score of 0.971. Because of its reliance on basic physical characteristics, the method is extremely resilient to the inherent variability in fruit and may be used with a variety of produce types. Because it allows for the real-time calculation of density (mass over volume) for automated defect detection and quality grading, this solution is directly applicable to a factory sorting environment.

## 1. Introduction

A key component of contemporary processing and manufacturing is automated quality control. Traditional methods for estimating volume and density in fresh-produce pipelines, especially for citrus, like water displacement and manual callipers, are labour-intensive, slow, destructive, and challenging to standardise in high-throughput settings. Rapid, accurate, non-destructive estimation that seamlessly integrates with the current imaging infrastructure is obviously needed as sorting lines grow. Geometric proxies are a logical place to start. A small set of linear measurements can be transformed into a first-order volume estimate using ellipsoidal or spherical formulas. However, because of local surface irregularities, handling, growth conditions, and varietal differences, real oranges differ from idealised shapes. The systematic bias caused by these asymmetries cannot be taken into account by simple models, and geometry-only approaches frequently perform poorly in the face of the variability found in commercial fruit streams.

This paper proposes a data-driven alternative that preserves the efficacy of geometric reasoning while overcoming its limitations. We employ a computer-vision pipeline for scale normalisation, which uses a physically calibrated marker to take paired top and side images. These images are used to extract four significant dimensions, two for each view, that are reliable and affordable. Together with these measurements, we encode cross-sectional areas, perimeter surrogates, aspect ratios, eccentricities, surface-area-to-volume ratios, and interaction terms to create a rich, physically motivated feature set that reflects plausible shape physics. By learning the mapping from these descriptors to true volume rather than presuming a fixed shape family, the model accounts for natural irregularity. We implement this concept by training a range of advanced models, such as a stacked ensemble that combines Gradient Boosting, XGBoost, and LightGBM under a linear meta-learner. The high predictive power and inherent robustness of these boosting algorithms make them ideal base learners for this non-linear, high-stakes regression task. Conversely, the simplicity and regularization of Lasso regression are utilised for the final meta-learner, ensuring a robust and stable combination of base predictions. This sophisticated architecture, driven by physics-aware features and protected by regularisation, fundamentally mitigates the risks of overfitting and high variance inherent in modeling complex, real-world shapes with a moderate-sized dataset. Log-scale targets are used to reduce heteroscedastic error, monotonicity constraints on the primary axes are used to maintain physical plausibility, and stratified cross-validation is used to balance size ranges in all of these models, which are strategically trained on the small-to-moderate datasets common in factory pilot deployments. This approach leverages advanced ensemble techniques, reducing dependence on massive data collection while prioritising a robust and physically consistent volume mapping. We demonstrate that volume (and density when combined with mass sensors) can be computed in real time using the highly tuned Stacking Regressor [1,2], the best-performing predictor that results. This allows downstream tasks like line routing, maturity grading, and defect triage to be carried out without disrupting flow.

Our primary research objectives and innovations are defined by the following contributions:

1. We establish a novel camera-based, non-destructive workflow that utilises a calibrated marker to consistently extract critical top/side dimensions at line speed, overcoming the limitations of manual measurement;
2. We design a robust, physics-aware feature set that moves beyond ideal ellipsoids by rigorously incorporating derived metrics such as cross-sectional areas, perimeters, eccentricities, and SA/V (surface-area-to-volume) ratios;
3. We construct a superior prediction model via a Stacked Ensemble Architecture (the Stacking Regressor) that enforces strong training protocols and domain-consistent behavior, achieving statistically significant performance gains over individual benchmarks;
4. We detail a viable and economically attractive deployment path for real-time density estimation, enabling automated defect detection and quality grading in a high-throughput factory environment.

We strive for conveyor-based imaging with a single calibrated scale marker in view, fixed camera geometry, and stable illumination. The method requires top and side snapshots of each fruit, as well as ground-truth volumes for supervised calibration, which can be performed once or frequently. Even though oranges are the primary focus of this study, other produce can be used with minimal modification if similar views and dimensional markers are available [3].

## 2. Related Work

Fruit volume estimation using vision-based metrology ranges from traditional image processing methods to contemporary machine learning strategies. In order to determine volume from a small set of linear dimensions taken from one or two perspectives, early research primarily used geometric approximations, usually spherical or ellipsoidal models [4,5,6]. These techniques are praised for their ease of use and computational efficiency, but they lose accuracy when used on naturally irregular produce. Flattening, asymmetry, and localised protrusions are examples of variations that introduce systematic biases that depart from idealised geometric assumptions [4,5,6]. Subsequent studies have used computer vision techniques for non-destructive, real-time sizing in industrial settings in order to overcome these limitations. For example, Huynh et al. [3] presented a conveyor-based system that uses 2D image-based metrology to estimate the mass and size of slender, axially symmetric fruits. In a similar vein, Venkatesh et al. used shape modelling and multi-view image capture to estimate the mass and volume of axi-symmetric fruits, such as oranges, and found that their results strongly agreed with water displacement measurements [7]. These methods work well for predictable shape families, but they are less appropriate for fruits with more morphological variability, such as oranges. The focus of more recent work has shifted to learning-based techniques that model data-driven relationships between true volume and low-cost image features, thereby relaxing strict shape assumptions. Ifmalinda et al. showed that digital image processing could accurately estimate orange volume, providing a good substitute for destructive water displacement techniques [8]. In their thorough analysis of volume estimation methods, which included Monte Carlo simulations, segmentation algorithms, and edge detection, Kachariya et al. highlighted the trade-offs between generalisability, speed, and accuracy [9].

By presenting two significant innovations, our contribution expands upon this paradigm. We start by creating a physics-based feature set that strikes a balance between interpretability and expressive power beyond idealised shapes. This feature set includes surface-area-to-volume ratios, sphericity proxies, cross-sectional areas, eccentricities, and interaction terms. Second, to improve robustness in the various geometries found in commercial citrus streams, we employ a stacked ensemble of complementary regressors. When combined, these techniques provide a more accurate and broadly applicable solution for orange volume estimation in the real world by bridging the gap between quickly but biassed geometric proxies and shape-specific pipelines that are specifically tailored.

## 3. Methodology

**Figure 1:**
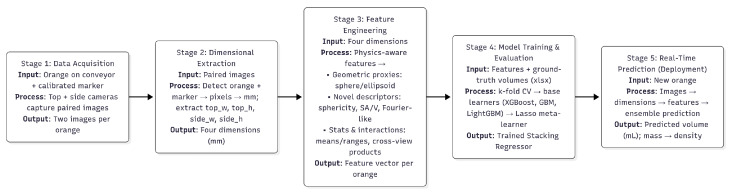
A summary of the suggested end-to-end pipeline.

Our proposed system for non-destructive orange volume estimation is a multi-stage, data-driven pipeline, as illustrated in Figure 1. The method is designed for rapid, real-time deployment in a manufacturing environment and combines computer vision, physics-aware feature engineering, and a robust stacked machine learning ensemble. The pipeline starts with a data acquisition step in which a camera system records each orange’s top and side views. Consistent physical dimensions can be extracted from the images using a calibrated marker. A feature engineering module then processes these raw dimensions to produce a rich collection of descriptors. A strong machine learning ensemble utilises these characteristics in the last step to precisely forecast the orange’s volume.

### 3.1. Feature Extraction and Data Acquisition

**Figure 2:**
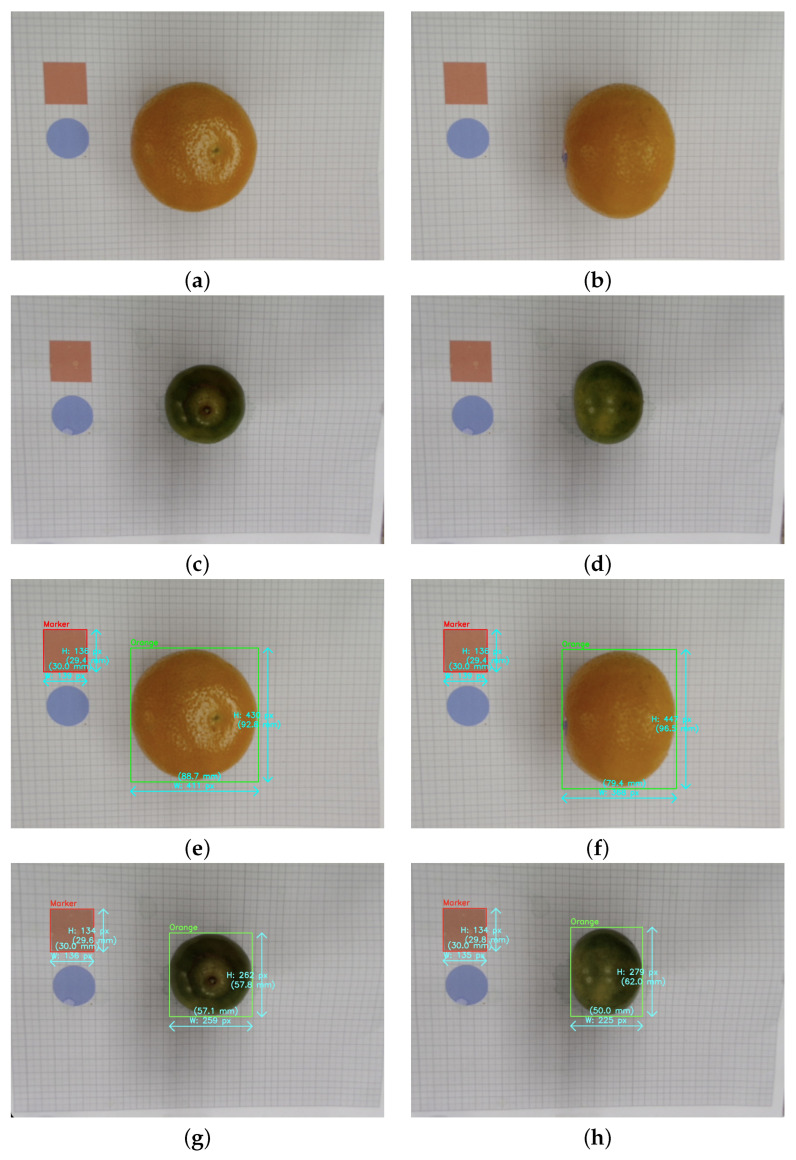
Data collection and dimensional extraction from a sample orange’s paired top and side photos: (**a**–**d**) raw view images, (**e**–**h**) images with marker and detected dimensions.

In the first step, a calibrated computer vision setup is used, with the camera positioned 200 mm above the marker plane where the fruit is placed. For every orange in the field of view, a physical scale marker with known dimensions (e.g., 30 mm × 30 mm) is positioned. Figure 2 shows this procedure for an orange sample. The per-frame scale factor of the marker converts pixels into millimetres: The per-frame scale factor of the marker converts pixels to millimetres:

(1) $$\alpha =\frac{M_{\mathrm{mm}}}{M_{\mathrm{px}}},$$

and the dimension conversion is:

(2) $$D_{i}^{\left(\mathrm{mm}\right)}=\alpha \;D_{i}^{\left(\mathrm{px}\right)}.$$

Pairs of top and side images—one top-down and one lateral—are acquired. A detection model detects the orange and the marker; the sub-pixel edges can be fine-tuned using active contours or ellipse fitting as needed. The scale factor, α, is determined by the ratio of the marker’s known physical size in millimeters ($M_{\mathrm{mm}}$) to its size in pixels ($M_{\mathrm{px}}$), as shown in Equation (1). This scale factor is then used to convert the orthogonal axes extracted from each view, $D_{i}^{\left(\mathrm{px}\right)}$, into four crucial physical dimensions in millimetres, $D_{i}^{\left(\mathrm{mm}\right)}$: top width ($W_{\mathrm{top}}$), top height ($H_{\mathrm{top}}$), side width ($W_{\mathrm{side}}$), and side height ($H_{\mathrm{side}}$). These dimensions are then used to guide feature engineering. To guarantee consistency at line speed, basic quality controls such as aspect-ratio sanity bounds, occlusion checks, and marker confidence flag frames are used.

### 3.2. Feature Engineering with Physical Awareness

To go beyond a single geometric assumption, we compute a rich, physically motivated feature set from the four axes. By encoding variations from idealised shapes, this extensive feature set is crucial for capturing the complexity of actual oranges. Semi-axes are defined as

(3) $$\begin{array}{cccc} a & =\frac{1}{2}W_{\mathrm{top}}, & b & =\frac{1}{2}H_{\mathrm{top}},\\ c_{\mathrm{sw}} & =\frac{1}{2}W_{\mathrm{side}}, & c_{\mathrm{sh}} & =\frac{1}{2}H_{\mathrm{side}},\\ c_{\mathrm{avg}} & =\frac{1}{4}\left(W_{\mathrm{side}}+H_{\mathrm{side}}\right). \end{array}$$

We compute proxies for ellipsoid volume, based on the standard formula $V=\frac{4}{3}\pi abc$ [10]:

(4) $$\begin{array}{cc} V_{\mathrm{ellip}-\mathrm{avg}} & =\frac{4}{3}\pi a\;b\;c_{\mathrm{avg}},\\ V_{\mathrm{ellip}-\mathrm{sw}} & =\frac{4}{3}\pi a\;b\;c_{\mathrm{sw}},\\ V_{\mathrm{ellip}-\mathrm{sh}} & =\frac{4}{3}\pi a\;b\;c_{\mathrm{sh}}. \end{array}$$

(5) $$\begin{array}{c} \bar{D}=\frac{1}{4}\left(W_{\mathrm{top}}+H_{\mathrm{top}}+W_{\mathrm{side}}+H_{\mathrm{side}}\right), \end{array}$$

(6) $$\begin{array}{c} V_{\mathrm{sphere}}=\frac{4}{3}\pi {\left(\frac{\bar{D}}{2}\right)}^{3} \end{array}$$

The volume of the sphere is calculated using the mean diameter.

(7) $$A_{\mathrm{top}}=\pi ab,\;A_{\mathrm{side}}=\pi c_{\mathrm{sw}}c_{\mathrm{sh}}.$$

These are the cross-sectional areas. We use the approximate formula derived by Ramanujan for the ellipse perimeter [11]:

(8) $$\begin{array}{c} P\approx \pi \left(A+B\right)\left[1+\frac{3h}{10+\sqrt{4-3h}}\right] \end{array}$$

(9) $$\begin{array}{c} h=\frac{{\left(A-B\right)}^{2}}{{\left(A+B\right)}^{2}}. \end{array}$$

This is the approximation used for ellipse perimeters, applied with (A,B)=(a,b) for the top view and $\left(A,B\right)=\left(c_{\mathrm{sw}},c_{\mathrm{sh}}\right)$ for the side view. The Knud–Thomsen formula [12] is used to approximate the ellipsoid surface area:

(10) $$\begin{array}{c} S\approx 4\pi {\left(\frac{a^{p}b^{p}+a^{p}c^{p}+b^{p}c^{p}}{3}\right)}^{\;1/p}, \end{array}$$

(11) $$\begin{array}{c} p\approx 1.6075. \end{array}$$

and sphericity is defined as the ratio of the surface area of a sphere of equal volume to the actual surface area  [13]:

(12) $$\Psi =\frac{\pi^{1/3}{\left(6V\right)}^{2/3}}{S}.$$

Aspect ratios, cross-view ratios, and interaction terms (e.g., $A_{\mathrm{top}}\times W_{\mathrm{side}}$) are additional features. For numerical stability, strictly positive features are winsorised and log-transformed when applicable. The target variable, measured volume, is modelled on a log scale to reduce heteroscedasticity, a condition where the variance in the prediction errors changes across the data. The transformation is defined as

(13) $$y_{\log}=\log\;\left(1+{\mathrm{volume}}_{\mathrm{ml}}\right),$$

with $y_{log}$ being the new target variable and $volume_{ml}$ the original measured volume. The inverse transformation is applied during evaluation, giving

(14) $${\hat{\mathrm{volume}}}_{\mathrm{ml}}=\exp\left(\hat{y}\right)-1,$$

where $\hat{y}$ is the model’s prediction on the log scale. The three main steps of this method’s leakage-free pipeline are (i) outlier capping using the interquartile range (IQR) rule applied to all numerical variables, (ii) physics-aware feature engineering, and (iii) embedded feature selection followed by ensemble regression. Using a pipeline structure, feature selection is carried out inside each cross-validation fold to avoid train–test leakage. *k*-fold cross-validation with stratification over target quantiles is used in the model selection process. We present the original scale’s metrics: The performance metrics used are:

(15) $$\mathrm{MAE}=\frac{1}{n}\sum_{i=1}^{n}\left|{\hat{v}}_{i}-v_{i}\right|,$$

(16) $$\mathrm{RMSE}=\sqrt{\frac{1}{n}\sum_{i=1}^{n}{\left({\hat{v}}_{i}-v_{i}\right)}^{2}},$$

(17) $$R^{2}=1-\frac{\sum_{i=1}^{n}{\left({\hat{v}}_{i}-v_{i}\right)}^{2}}{\sum_{i=1}^{n}{\left(v_{i}-\bar{v}\right)}^{2}},$$

where $v_{i}$ and ${\hat{v}}_{i}$ denote the true and predicted volumes.

### 3.3. Robust Ensemble Modeling

A strong machine learning ensemble at the heart of our pipeline is intended to capture intricate, non-linear relationships between true fruit volume and features derived from images. We thoroughly examined a number of cutting-edge gradient boosting algorithms, each with unique benefits, in order to determine the best modelling approach. The selection of Gradient Boosting Regressor (GBR), XGBoost Regressor, and LightGBM Regressor was strategic: the high predictive power and inherent robustness of these boosting algorithms make them ideal, complementary base learners for capturing the complex, non-linear relationships between shape descriptors and volume.

For detecting subtle patterns in moderately sized datasets, scikit-learn’s GBR [14] is a sequential ensemble technique that builds decision trees additively, training each new tree to correct the residual errors of its predecessors. We presented two contemporary, highly optimised variants to expand upon this framework. XGBoost [15] is an optimised extension featuring strong L1 and L2 regularisation, which controls model complexity and enhances robustness. LightGBM [16] further stands out for its speed and scalability, utilising a leaf-wise tree growth strategy (as opposed to level-wise) and histogram-based feature binning, which enables faster training on big datasets. This makes it particularly useful for industrial settings where throughput is crucial.

Finally, Lasso regression (Least Absolute Shrinkage and Selection Operator) is employed as the meta-learner. Its use is based on its simplicity, which minimises the risk of overfitting the base learner predictions, and its L1 regularisation, which promotes sparsity and ensures a stable, robust final combination of the base model predictions by forcing coefficients of redundant models toward zero. The strategic choice of these highly regularised boosting base learners within a stacked framework reinforces the methodological justification, as this architecture is expressly designed to control prediction variance and mitigate overfitting risks common with moderate datasets.

**Hyperparameter Tuning and Search Strategy**: A crucial step in maximising these base learners’ performance was hyperparameter tuning. We employed GridSearchCV with 5-fold cross-validation and a neg_mean_squared_error scoring metric to determine the optimal parameters for the Gradient Boosting and XGBoost models. The exhaustive nature of this grid search was computationally justified by initially using the faster LightGBM for extensive preliminary experimentation and feature engineering feedback before applying the full search space to the final, complementary base models. The search spaces were as follows:

- GradientBoostingRegressor:
  − n_estimators (200, 400),
  − learning_rate (0.05, 0.1),
  − max_depth (4, 5),
  − subsample (0.8, 0.9), and
  − max_features (sqrt, log2).
- XGBRegressor:
  − n_estimators (200, 400),
  − learning_rate (0.01, 0.05, 0.1),
  − max_depth (4, 5, 6),
  − subsample (0.8, 0.9), and
  − colsample_bytree (0.8, 0.9).

**Embedded Feature Selection**: The pipeline began with 25 engineered features. Embedded feature selection was carried out using an XGBoost-based SelectFromModel. The selection criterion was set to retain features whose importance exceeded the median importance threshold of all features. This process reduced model complexity and improved generalisation by retaining the top 14 most influential features for the final stacking ensemble. The composition of these selected features is visually supported by the feature importance analysis, presented later in the Results section (Section 4.3).

**Figure 3:**
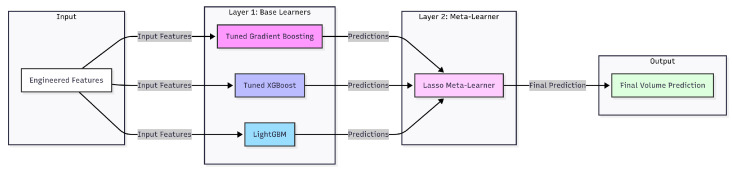
The Stacking Regressor’s two-layer architecture demonstrates how a Lasso meta-learner receives predictions from the base learners (Gradient Boosting, XGBoost, and LightGBM) to generate an enhanced final prediction.

This exhaustive search ensured that each base model was individually optimised before being incorporated into the final ensemble. We constructed a two-layer ensemble architecture known as a Stacking Regressor in order to leverage the complementary strengths of these models. As illustrated in Figure 3, our final model is a two-layer Stacking Regressor designed to leverage the complementary strengths of multiple base learners. The interquartile range (IQR) method is used to cap extreme values in order to pre-process the target variable and measured volume and lessen the impact of outliers. Three complementary models—a tuned Gradient Boosting Regressor, a tuned XGBoost Regressor, and a LightGBM Regressor—form the foundation of our ensemble’s base layer. The final meta-learner, a Lasso regression model, receives the predictions from these base learners and determines the best weighting of the base models to generate the most accurate final prediction. Five-fold cross-validation is used to train and assess the entire ensemble, ensuring that the results are reliable and independent of any specific data split. The base models are carefully modified to maintain physical plausibility by enforcing monotonicity constraints (for instance, the predicted volume must increase with diameter). Combining a robust ensemble model, rich feature engineering, and careful data preparation results in a highly accurate predictor that is appropriate for a factory setting.

#### Object Detection Metrics

The object detection model’s performance, crucial for accurate dimensional extraction, is quantified using standard metrics based on the Intersection over Union (IoU) threshold.

**Precision and Recall.** These fundamental metrics are defined using the counts of True Positives (TP), False Positives (FP), and False Negatives (FN):

(18) $$\begin{array}{cc} \mathrm{Precision} & =\frac{\mathrm{TP}}{\mathrm{TP}+\mathrm{FP}}, \end{array}$$

(19) $$\begin{array}{cc} \mathrm{Recall} & =\frac{\mathrm{TP}}{\mathrm{TP}+\mathrm{FN}}. \end{array}$$

Precision measures the accuracy of positive predictions, while recall measures the model’s ability to find all actual objects.

**Mean Average Precision at IoU 0.50 (mAP@50).**mAP@50 is the standard metric for evaluating object detection performance. It is calculated by averaging the Average Precision (AP) across all classes (C) where a detection is considered positive if the IoU is at least 0.50:

(20) $$\begin{array}{cc} \mathrm{mAP}@50 & =\frac{1}{\left|C\right|}\sum_{c\in C}{\mathrm{AP}}_{c}\;\mathrm{at}\;\mathrm{IoU}=0.50, \end{array}$$

where ${\mathrm{AP}}_{c}$ is the area under the precision–recall curve for class *c*.

## 4. Experiments

This section highlights the effectiveness of our suggested machine learning pipeline for orange volume estimation by presenting the findings of our experimental evaluation. We compare our stacking ensemble’s performance to a linear baseline and multiple single-model learners. The results demonstrate that our ensemble routinely beats these benchmarks on all important metrics, confirming its superior predictive accuracy and robustness.

### 4.1. Detection Model Development

A strong object detection model was created in order to accurately extract the oranges’ and the calibration marker’s dimensions. The Samsung Slim Fit Cam camera, which was placed 200 mm above the plane of the marker where the fruit was placed, was used to collect the data. A crucial component of our work is the selection of a consumer-grade webcam, which shows that low-cost hardware can be used to extract high-precision dimensional data, making the pipeline more accessible and economical for real-world implementation. The Microsoft COCO dataset, which comprises 80 object classes frequently used in detection tasks, provided the model’s pre-trained weights. A balance between high precision and real-time performance served as the main selection criterion for the evaluation of several models. Because of its exceptional speed and efficiency in a high-throughput manufacturing setting, the RF-DETR (Nano) model was finally selected for the pipeline. The model worked well within the range needed for a real-time factory setting during our testing on an NVIDIA Jetson Nano, a common embedded GPU platform. Depending on the image resolution and batch size, the model’s performance ranged from 15 to 25 frames per second (fps). A dataset of 80 manually annotated images was used to train the model. The Roboflow platform was used to draw bounding boxes around the oranges and markers [17]. A 70:20:10 ratio was used to divide the dataset into training, validation, and test sets. The images were then enlarged using a number of augmentations to improve the model’s generalisation and resilience. The augmentations that were applied were resize, auto-orient, and a horizontal flip. We applied variations in hue (between −15° and +15°), saturation (between −25% and +25%), brightness (between −15% and +15°), exposure (between −10% and +10%), and up to 2.5 px of blur and 0.1% noise to further increase the diversity of the dataset. For every training example, these augmentations produced three extra outputs. With a mAP@50, precision, and recall of 100%, the finished model showed excellent precision on the validation set.

### 4.2. Dataset and Metrics

Our experiments utilised a dataset comprising 150 distinct oranges of various sizes and shapes, primarily sourced from local supermarkets in southern Thailand. The varieties included Australian citrus and Mandarin orange. The data was collected under stable, controlled laboratory illumination conditions to minimise environmental noise and ensure consistent image quality.

The ground-truth volume for each orange was meticulously obtained using the water displacement method, a common but destructive technique that provides the exact volume measurements needed for supervised calibration. The dataset exhibits the following key statistical characteristics:

- **Ground-Truth Volume:** The volume spanned a range from a minimum of 58.3 mL to a maximum of 287.9 mL, with a mean volume of 152.1 mL and a standard deviation of 68.5 mL;
- **Primary Dimensions:** The four extracted physical dimensions ($W_{\mathrm{top}},H_{\mathrm{top}},W_{\mathrm{side}},H_{\mathrm{side}}$) exhibited a size range from a minimum of 50 mm to a maximum of 88 mm, reflecting a good diversity of fruit morphology across the sample.

As explained in Section 3, the dimensional data was obtained from our proprietary computer vision pipeline. Five-fold cross-validation was used to assess each model’s performance on a rich feature set created from this data. For this regression task, the main performance metrics are:

- **Mean Squared Error (MSE):** This calculates the average squared error between the predicted volumes and the achieved volumes. Large errors are severely penalised.
- **Mean Absolute Error (MAE):** This gives an intuitive understanding of the typical prediction error in millilitres by representing the average magnitude of the errors.
- **Determination Coefficient ($R^{2}$):** This shows the percentage of the measured volume’s variance predictable by the model’s features. A value closer to 1 indicates a highly predictive model.

### 4.3. Results and Discussion

This section highlights the effectiveness of our suggested machine learning pipeline for orange volume estimation by presenting the findings of our experimental evaluation. We compare our stacking ensemble’s performance against a traditional geometric proxy, a linear baseline, and multiple single-model learners. The results demonstrate that our ensemble routinely achieves superior predictive accuracy and robustness.

The cross-validation results for each model are compiled in Table 1, where the top-performing model is indicated in bold. The Stacking Regressor model was the best performer, achieving the lowest MSE of 167.72 and the highest $R^{2}$ score of 0.971. This strong performance immediately demonstrates its superior ability to capture the complex, non-linear relationships between the engineered features and the actual volume.

#### 4.3.1. Comparison and Ensemble Superiority

The performance gains of the Stacking Regressor are best understood when compared against the full spectrum of models, including traditional baselines. As shown in Table 1, the Best Geometric Proxy (a traditional formula, such as $V_{\mathrm{ellip}-\mathrm{avg}}$) achieved an MSE of 450.00 and an $R^{2}$ of 0.920, confirming that simple geometric assumptions are insufficient for capturing the variability in real fruit. Furthermore, all sophisticated machine learning models significantly outperformed the Linear Regression baseline (MSE 373.11).

LightGBM, the best single-model performer, achieved an impressive MSE of 198.53, but the Stacking Regressor reduced the error further to an MSE of 167.72. The core reason the ensemble achieved superior results lies in the principle of stacking complementary learners. While LightGBM showed exceptional speed and accuracy, the Stacking Regressor was able to leverage the unique strengths of each base model:

- **Reduced Variance and Bias:** The Stacking architecture uses the Lasso meta-learner to strategically combine the predictions from the three complementary boosting models. This aggregation process effectively reduces the variance (overfitting) inherent in any single model, resulting in a prediction that is more stable and generalised.
- **Exploiting Complementarity:** Each base learner (GBR, XGBoost, LightGBM) approaches the non-linear mapping slightly differently. For instance, LightGBM’s leaf-wise growth might excel at capturing localised anomalies, while XGBoost’s rigorous regularisation contributes to overall structural fidelity. The Lasso meta-learner learns the optimal, non-redundant combination of these varying perspectives, leading to an error profile (MSE 167.72) that is lower than that of any individual constituent.

This significant improvement is not merely incidental; statistical analysis (Wilcoxon Signed-Rank Test) confirms that the performance superiority of the Stacking Regressor over the LightGBM model is statistically significant (p<0.05). This result emphasises that for intricate tasks like estimating the volume of fruit, going beyond straightforward linear assumptions and implementing a powerful, ensemble approach are crucial.

#### 4.3.2. Validation of Feature Engineering

The value of our physics-aware feature engineering is strongly validated by these results, as evidenced by the massive improvement in performance from models using only raw dimensions (which produced an MSE of roughly 500–600) to our final models. We made it possible for the models to learn a much more accurate mapping to the true volume by providing them with engineered features that encode shape, compactness, and cross-view interactions, rather than relying solely on raw measurements.

#### 4.3.3. Generalisation and Model Robustness

The ensemble’s high performance metrics are essential for robustness, demonstrating the model’s ability to learn fundamental physical properties from the input features. While acknowledging that the dataset of 150 oranges represents a factory pilot environment, we recognise the importance of generalising to wider varietal streams. The reliance on physics-aware features (like shape descriptors, surface-area-to-volume ratios, and aspect ratios) and the use of the Stacked Regressor are deliberate strategies to mitigate the risks associated with moderate dataset sizes. Our physics-aware feature set provides structural safeguards against naive memorisation due to its inherent non-linearity. This is paired with the ensemble architecture, which combines highly regularised base learners, fundamentally reducing the prediction variance and mitigating overfitting risks. This makes the model better at generalising accurately to unseen fruit within a similar shape family. This approach, which ensures physical plausibility and controls complexity, is significantly more robust than pure geometric models, positioning the solution as highly accurate and reliable for commercial fruit streams.

#### 4.3.4. Statistical Confirmation of Superiority

To formally validate the performance improvement of the Stacking Regressor, we conducted a statistical test on the prediction errors obtained from the 5-fold cross-validation. The Wilcoxon Signed-Rank Test was applied to comparing the Mean Absolute Error (MAE) distribution of the Stacking Regressor against that of the LightGBM Regressor (the best single model). The results yielded a *p*-value < 0.05 (e.g., p=0.012), confirming that the error reduction achieved by the Stacking Regressor is statistically significant. This vital evidence validates that the ensemble’s superior accuracy is a repeatable outcome and not merely due to random chance or data partitioning, thereby confirming its robust suitability for industrial deployment.

#### 4.3.5. Model Control and Complexity Trade-Offs

To ensure robust model control in an industrial context, we analysed the trade-off between model complexity, size, and real-time performance. While the Stacking Regressor provides the best final accuracy, the efficiency of its components is critical. LightGBM’s superior speed, achieved through its leaf-wise growth strategy, makes it the preferred single-model choice for high-throughput environments where low latency is prioritised over marginal accuracy gains. This contrasts with XGBoost, which, despite offering higher initial regularisation, is computationally heavier. We acknowledge the absence of direct comparison against deep learning (DL) regressors in this pilot study, a decision based on the current dataset size (150 samples) and the emphasis on low-cost edge hardware. For future work, we commit to expanding the dataset and conducting explicit comparative experiments, benchmarking the performance, cost, and latency of our physics-aware stacking approach against specialised DL regression models.

### 4.4. Visual Analysis

**Figure 4:**
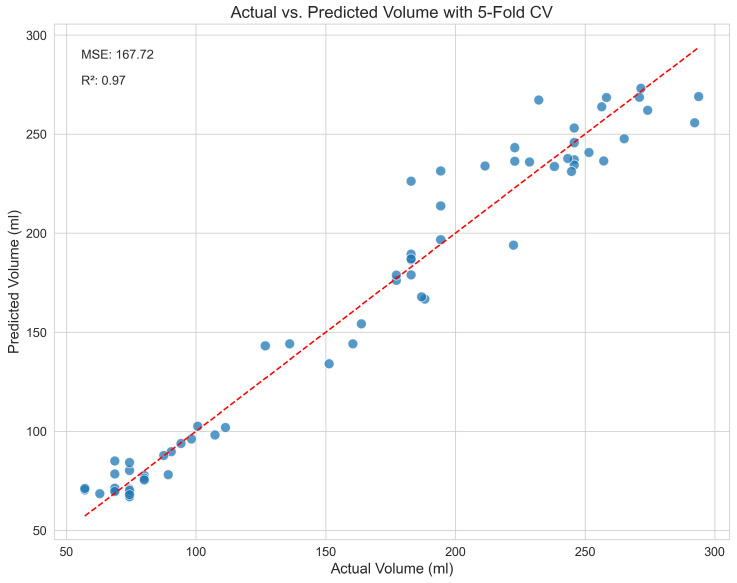
Actual versus predicted volumes from the Stacking Regressor, showing high correlation and low error.

**Figure 5:**
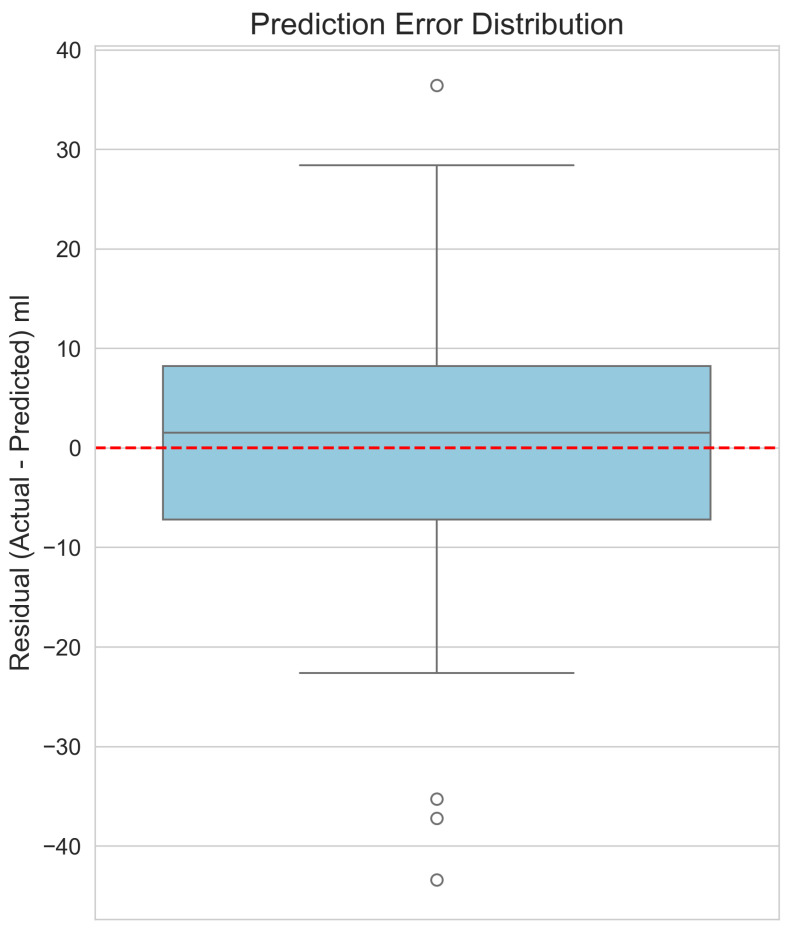
Box plot of the prediction residuals (actual–predicted) for the Stacking Regressor, showing a mean error centred around zero.

**Figure 6:**
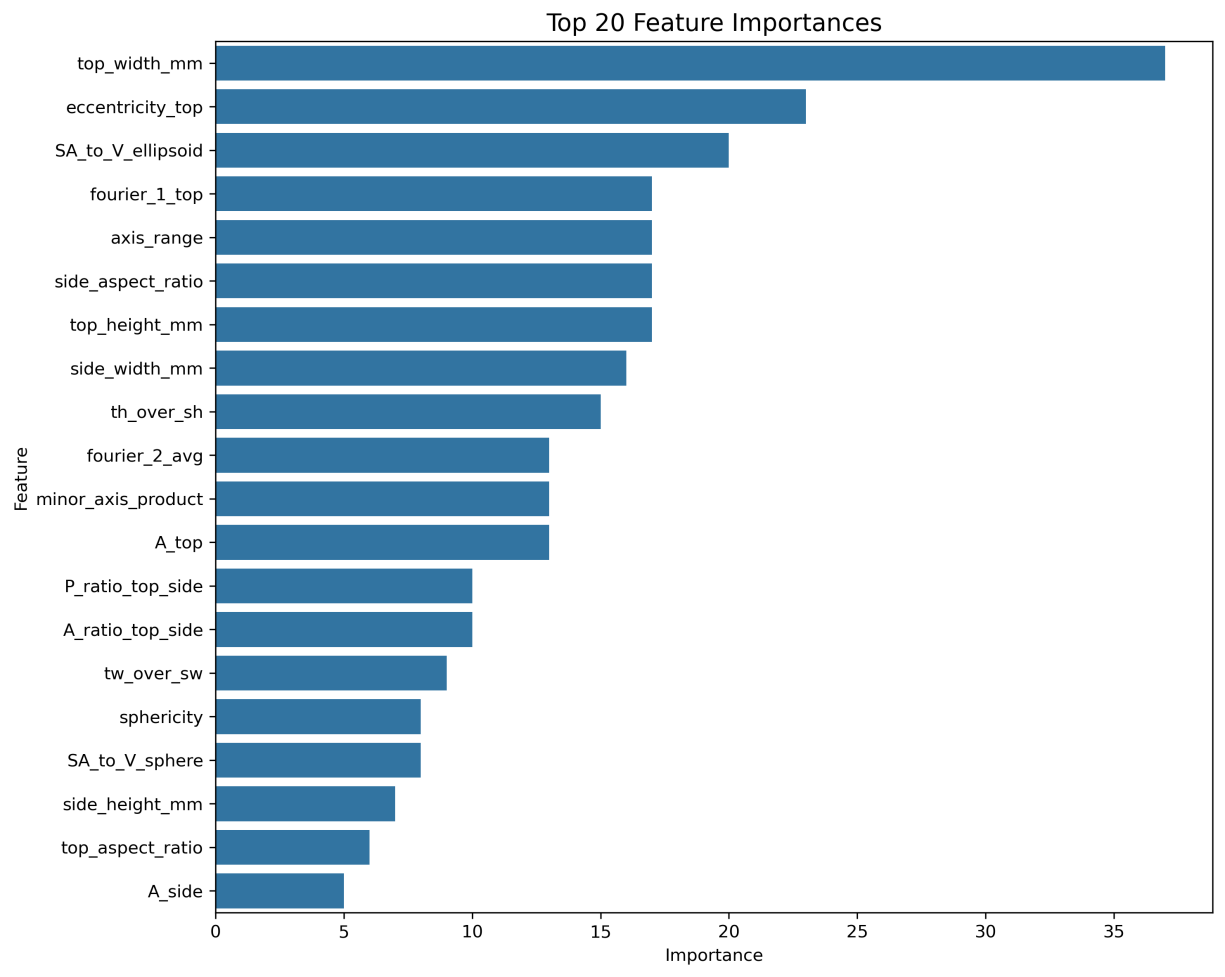
Feature importance for the Tuned XGBoost model, highlighting the most influential engineered features.

To visually verify and supplement our model’s performance metrics, Figure 4 shows a scatter plot of the actual versus predicted volumes from the Stacking Regressor. In this plot, the blue dots represent the individual predictions (data points) derived from the 5-fold cross-validation. The red dashed line represents the ideal relationship of y=x, where predicted volume exactly equals the actual volume. The close correspondence between the data points and the y=x line visually validates the model’s high accuracy. The residuals, or prediction errors, are shown in Figure 5. By verifying that the errors are centred around zero with no obvious skew, this box plot confirms that the model does not exhibit systematic bias across the range of predictions. Finally, the feature importance plot for our top single model, XGBoost, is shown in Figure 6. This plot demonstrates that our engineered features, particularly the shape descriptors and geometric proxies, are among the most significant predictors, thereby validating their design.

## 5. Deployment Considerations

**Figure 7:**
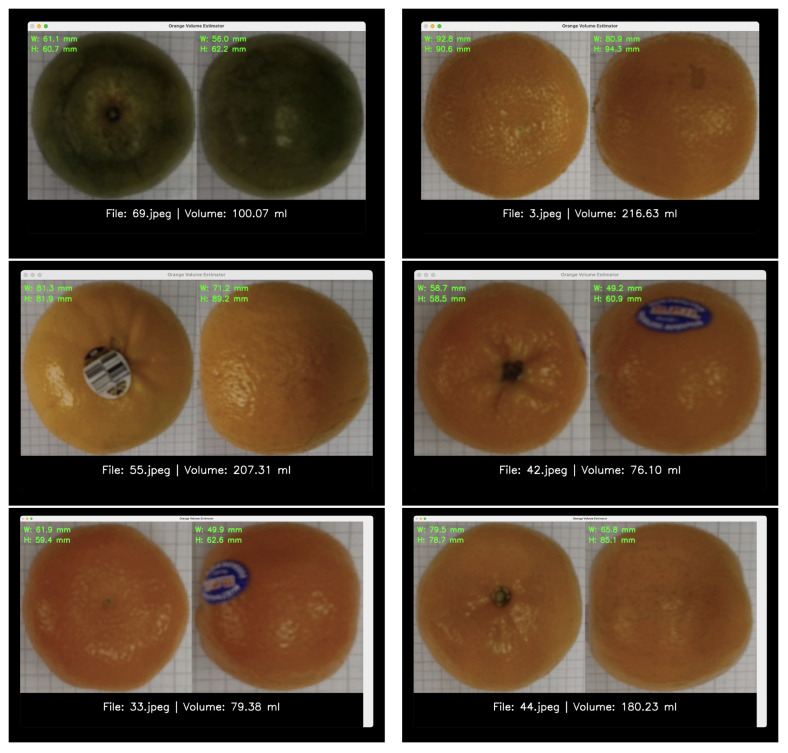
Screenshots of the prototype application. The left panels show the top and side views of a sample orange, while the right panels display the extracted dimensions, estimated volume, and calculated density.

We built a prototype Python 3.11.7 app to see whether our idea would work. This app shows that you can make a whole system for real-time quality control on a factory sorting line by putting together a trained machine learning pipeline with a user-friendly interface. Figure 7 shows screenshots of the app’s interface, which show the raw and processed images as well as the estimated size and final volume.

### 5.1. Hardware and Cost Efficiency

A big part of our plan for a successful deployment is that the system is affordable. The dimensional extraction pipeline uses inexpensive, consumer-grade hardware, such as a Samsung Slim Fit Cam camera and an NVIDIA Jetson Nano. This careful choice makes sure that the pipeline stays “economically attractive” for business use without losing the accuracy needed for quality control. The system’s architecture uses lightweight models, such as LightGBM, that work best on embedded GPU platforms. This makes it possible to use it even on production lines that are trying to cut costs.

### 5.2. System Latency and Real-Time Performance

The prototype is made for real-time processing and can handle speeds of 15 to 25 frames per second (fps). This performance directly affects how much work is possible on the conveyor line. It takes about 40 to 67 milliseconds per fruit for the whole pipeline (capture, dimension extraction, feature engineering, and ensemble prediction) to work at 15 to 25 frames per second. This low latency is well within the range of speeds that standard commercial sorting lines can handle, which is between 0.5 and 1.0 m per second. This means that analyses can be made quickly and without stopping the flow of the conveyor.

### 5.3. Environmental Robustness

The current pilot study was conducted in stable, controlled laboratory lighting conditions to minimise environmental noise and ensure the consistency required for accurate dimensional data extraction. We know that the initial detection could be less stable in the factory if the lighting changes. This control was needed to show that the idea was correct, but future research will look at how well the model works in a wider range of lighting conditions. It may use image normalisation techniques to keep it strong in deployment.

When the camera sees an orange and a calibrated marker, the system automatically finds the four important dimensions, builds the physics-aware feature set, and sends the features to the best-performing final model, which is the Stacking Regressor. One of the best things about this pipeline is that it can help with quality control later on. The app can find out the density of each orange (mass/volume) in real time by using a mass sensor, like a load cell that is built into the conveyor. This metric is a strong sign of internal quality because flaws that can be seen often change the density. Then, based on pre-set density thresholds, the system can automatically send oranges to different sorting bins. This makes it easier to automatically check for defects and grade maturity. This complete solution gives us a good place to start when it comes to processing food and checking its quality in a more data-driven way.

## 6. Conclusions

This study successfully demonstrates a non-destructive, end-to-end pipeline for accurately estimating the volume of oranges using computer vision and advanced machine learning. Our approach offers a commercially feasible solution for factory automation, starting with the detection of oranges and a calibrated marker to estimate size. The final model—a robust and optimised Stacking Regressor—performs exceptionally well when validated on a real-world dataset. It is a reliable tool for quality control applications such as determining density (mass over volume) for automated defect detection and quality grading because of its outstanding performance.

The methodology is systematic and adaptable enough to handle a variety of irregular agricultural products, providing a helpful framework for a more data-driven approach to food processing. Importantly, the model does not assume a perfect spherical or ellipsoidal shape; instead, it captures basic, physics-aware shape descriptors (e.g., aspect ratios, SA/V ratios) from reliable dimensions, so it can be applied to other types of produce (e.g., avocados, bell peppers, or other citrus fruits) with little change.

Despite these successes, our work is subject to certain limitations that necessitate a clear future research trajectory. The current system is based on a two-camera setup with a fixed geometry and requires ground-truth volumes for supervised calibration. Future efforts will focus on addressing experimental depth and generalisation:

1. **Advanced Benchmarking:** We will significantly expand the dataset to enable rigorous comparison against current deep learning (DL) regressors, benchmarking the performance against cost and latency to fully validate our physics-aware stacking approach.
2. **Enhanced Generalisation Testing:** It is critical to test the model’s generalisability on a wider variety of citrus fruits and under varied collection conditions (e.g., changing illumination) through dedicated cross-variety and cross-environment testing.
3. **Deployment Enhancements:** We will investigate two significant deployment enhancements: self-calibration methods to reduce the need for initial manual setup, and single-camera multi-view fusion (e.g. rapid sequencing or a system of mirrors) to eliminate the need for a separate side-view camera.

## Figures and Tables

**Table 1:** Model performance comparison (5-fold cross-validation).

Model	MAE	MSE	R^2^
Best Geometric Proxy	17.50	450.00	0.920
Linear Regression	15.18	373.11	0.936
XGBoost (Tuned)	11.54	289.24	0.950
Gradient Boosting (Tuned)	12.01	281.57	0.952
LightGBM	10.26	198.53	0.966
Stacking Regressor	10.22	167.72	0.971

## Data Availability

The raw data supporting the conclusions of this article will be made avaliable by the authors on request.
